# The Sanitarian

**Published:** 1880-12

**Authors:** 


*The Sanitarian.*—

This monthly publication devoted to the preservation of the health, and mental and physical culture, contains information important and interesting to every class of society. Whatever thing, condition or circumstance in rapport with, or antagonistic to, the most perfect culture of mind and body is considered legitimate matter for discussion, &c., in *The Sanitarian*, and its mission is the protection of human life. Terms: $3.00 a year, single number 30 cents. Editor and Publisher: A. M. Bell, A. M., M. D. 8 Spruce St., New York.

